# Determinants of Internet Addiction among Adolescents: A Case-Control Study

**DOI:** 10.1100/tsw.2011.85

**Published:** 2011-04-19

**Authors:** Artemis Tsitsika, Elena Critselis, Amalia Louizou, Mari Janikian, Aliki Freskou, Evgenia Marangou, Georgios Kormas, Dimitrios A. Kafetzis

**Affiliations:** ^1^ Adolescent Health Unit (A.H.U.), Second University Department of Pediatrics, “P. & A. Kyriakou” Children's Hospital, National and Kapodistrian University of Athens School of Medicine, Greece; ^2^ Second University Department of Pediatrics, “P. & A. Kyriakou” Children's Hospital, National and Kapodistrian University of Athens School of Medicine, Greece

**Keywords:** addictive behavior, Internet, adolescent, adolescent behavior, mental disorders

## Abstract

Internet Addiction (IA) is associated with adverse psychosocial development and mental disorders. The study aims were to evaluate the psychosocial profiles and psychiatric comorbidities associated with IA among adolescents. A case-control study was conducted among 129 adolescents in the outpatient setting of the Adolescent Health Unit of the Second University Department of Pediatrics in Athens, Greece. The case group consisted of 86 adolescents with IA as evaluated following psychiatric interview with two independent examiners. The control group consisted of 43 adolescents without IA, frequency matched for age and gender with case group participants. The study findings indicated that adolescents with IA were significantly more likely to have divorced parents (*p* = 0.012) and/or dysfunctional familial relationships (*p* < 0.0001). The proportion of adolescents with poor academic performance (*p* < 0.0001) and unexcused school absences (*p* = 0.004) was greater among those with IA. Moreover, approximately two-thirds of the adolescents with IA were engaged in high-risk behaviors (*p* < 0.0001). Finally, adolescents with IA were 3.89 times more likely to present with comorbid psychiatric conditions (CI 95%: 1.19–12.70), including depression (10.5 vs. 0%; *p* = 0.022). Adolescent IA is associated with deterred familial functions, poor academic performance, engagement in high-risk behaviors, and an augmented likelihood for depression.

## INTRODUCTION

The Internet has developed as the primary medium for communication and socialization, particularly among adolescents[1]. However, excessive Internet use may detrimentally affect academic performance, family relationships, and emotional development among youth. Such problematic Internet use has been identified as Internet Addiction (IA)[2] and related diagnostic criteria have been proposed[3]. Adolescents, in particular, are observed to be at enhanced risk for developing such behavioral patterns[4,5] since they are ever-increasingly exposed to the Internet without having fully developed their critical thinking skills and/or senses of boundaries[6].

IA has been proposed as a novel entity relating to behavioral addictive patterns of online and/or offline computer use[7] similar to those belonging within the spectrum of impulse-control disorders[8]. IA encompasses an uncontrollable use of the Internet, which is markedly distressing, time-consuming, and/or resulting in social and/or functional difficulties, and not solely present during hypomanic or manic clinical episodes[7]. In particular, to date, the proposed criteria for IA include the presentation of at least five of the six following criteria: (1) spending an increasing amount of time online, (2) failure to reduce use with concomitant feelings of restlessness and depression, (3) staying online longer than originally intended, (4) running the risk of losing a relationship or other opportunities due to Internet use, (5) lying to conceal the extent of Internet use, and (6) using the Internet in order to escape negative feelings[9].

The prevalence rate of IA among adolescents is reported to range between 1.00 and 18.3% in European countries[10,11,12,13], and between 13.7 and 18.4% in Asian countries[14,15]. In Greece, in particular, the prevalence rate of IA is observed to be 1%[10]. However, variability in the observed prevalence rates of IA among adolescents may be partly attributed to the fact that a consistent definition of IA and its assessment has not been established throughout the literature.

IA has been associated with numerous adverse psychosocial and mental health conditions. Specifically, maladaptive Internet use has been associated with the concomitant presence of dysfunctional and/or problematic family relationships[16]. In addition, adolescents with IA are more likely to have poor academic performance[17], limited extracurricular activities, and engagement in high-risk behaviors[18,19]. Finally, IA among adolescents is associated with the presentation of emotional problems and concomitant psychiatric conditions, including attention deficit hyperactivity disorder (ADHD)[20,21], depression, and suicidal ideation[15,22,23,24]. Therefore, it is posited that IA development may arise from composite interactions and events within and between the framework entities of personal, family, and psychosocial characteristics of adolescents. However, to date, a comprehensive assessment of those psychosocial and mental health conditions associated with IA and their etiological association have not been elucidated[25]. Moreover, the comparative contribution of personal, family, and psychosocial characteristics to the development of IA also remains obscure.

The primary objective of the present study is to assess the personal, clinical, family, and social characteristics of IA among adolescents. The secondary objective is to evaluate the predictive factors of IA in adolescents.

## METHODS

### Study Design and Study Population

A frequency-matched case-control study design was conducted. The study population consisted of adolescents attending the Adolescent Health Unit of the "P. & A. Kyriakou" Children's Hospital between June 1st, 2007 and June 1st, 2010. The study protocol was approved by the Ethical Committee of the "P. & A. Kyriakou" Children's Hospital in Athens, Greece. Informed consent for study participation was requested from the parents of all study participants prior to the initiation of the investigation.

The case group (n = 86) consisted of all adolescents who visited the setting during the study period and presented with IA. Assessment of IA was conducted through individual interviews with the Unit's health professional staff (including a pediatrician, a psychiatrist, and a clinical psychologist) blinded to the study objectives and was based on the proposed diagnostic criteria[9]. No exclusion criteria for participation in the present study were applied. The control group (n = 43) consisted of adolescents who visited our unit during the same period of time for reasons other than those of either excessive Internet use or IA. Case group subjects were selected following frequency matching according to gender and age with the case group population.

## Data Collection

In order to minimize any potential bias, two independent examiners evaluated all study subjects. The database consisted of six major components: (1) demographic information, (2) family structure and dynamics, (3) school performance and extracurricular activities (sports, hobbies), (4) psychosocial and clinical profile, (5) severity of IA, and (6) awareness of the problem.

Participants' age, gender, socioeconomic status, and highest parental educational level attained were included in the demographic features. The number of siblings, parents' marital status, as well as within family relationships (dysfunction in terms of communication, lack of boundaries, frequent conflicts, or harmonic relationships), were evaluated. Current academic achievement and school attendance, including unexcused school absences, were also evaluated. Participation in extracurricular activities and adoption of high-risk behaviors were assessed.

The psychosocial profile of adolescents was assessed by a psychiatrist and clinical psychologist blinded to the study objectives and participants' membership in the study. Individual interviews were conducted by the psychiatrist and clinical psychologist with each study participant on-site upon his/her first visit to the study center. The psychological variables examined included the presence of emotional problems, including anxiety, phobias, obsessions, depressive symptoms, weak social skills, and apathy. In addition, the concomitant presence of other psychiatric disorders (e.g., depression and ADHD) was also assessed according to the DSM-IV criteria. Finally, the presence of IA was also assessed during the aforementioned interviews according to established criteria[9].

## Statistical Analysis

Continuous variables are presented with mean and standard deviations, while categorical variables are presented with absolute and relative frequencies. For the comparisons of proportions, Chi-square and Fisher's exact tests were used. Student's t-tests were computed for the comparison of mean values. Stepwise multivariate logistic regression analysis was applied in order to evaluate the factors associated with the presence of IA (vs. non-IA) among adolescents. The independent factors entered into the multivariate regression model included personal, family, and academic characteristics, as well as presence of other comorbid psychiatric conditions. Adjusted odds ratios (AOR) with 95% confidence intervals (95% CI) were computed accordingly and the model fit was evaluated with the Hosmer and Lemeshow statistic. The predefined control group was applied as the basis of comparison for all statistical analyses undertaken. All $p$ values reported are two-tailed and the criterion for statistical significance was $p < 0.05$. All analyses were conducted using the SAS software program (version 9.2).

## RESULTS

Data from 129 participants (86 IA adolescents and 43 controls) were analyzed. The mean age for adolescents with IA was 15.3 years (SD = 1.6 years) and ranged from 11 to 18 years, while the mean age for those without IA was 15.2 years (SD = 1.4 years) and ranged from 12 to 18 years ($p = 0.627$). Table 1 presents the demographic characteristics for the two groups. The majority of adolescents with IA were male (Table 1). The two groups were similar in terms of area of residence and socioeconomic status. Moreover, the highest educational level attained by participants' parents were similar between the groups examined (Table 2).

**TABLE 1**
**Demographic Characteristics of the Adolescent Study Population According to the Presence of IA (n = 129)**

| | Case Group (n = 86) n (%) | Control Group (n = 43) n (%) | Chi-Square *p* Value |
|---|---|---|---|
| Gender | | | |
| Girls | 4 (4.6) | 4 (9.3) | 0.172* |
| Boys | 82 (95.4) | 39 (90.7) | |
| Age (mean years ± SD) | 15.3 ± 1.6 | 15.2 ± 1.4 | 0.627† |
| School grade | | | |
| Junior high school | 20 (23.3) | 10 (23.3) | 1.000 |
| High school | 66 (76.7) | 33 (76.7) | |
| Area of residence | | | |
| Suburban | 10 (11.6) | 5 (11.6) | 1.000 |
| Urban | 76 (88.4) | 38 (88.4) | |
| Socioeconomic status | | | |
| Low | 7 (8.1) | 6 (14.0) | 0.139* |
| Middle | 61 (70.9) | 32 (74.4) | 0.677 |
| High | 18 (20.9) | 5 (11.6) | 0.193 |

SD: standard deviation

* Fisher's exact test *p* value.

† T-test *p* value.

Table 2 presents the comparison of family characteristics among study participants according to the presence of IA. The proportion of adolescents having divorced parents was significantly greater among those with IA (25.6 vs. 7.0%, $p = 0.012$). In addition, the proportion of adolescents reporting harmonic family relationships was significantly lower among those with IA (14.0 vs. 74.4%, $p < 0.0001$). In contrast, the proportion of those reporting either dysfunctional (47.7 vs. 0.0%, $p < 0.0001$) or problematic (20.9 vs. 0.0%, $p = 0.001$) family relations were significantly greater among adolescents with IA.

Comparisons of academic attendance and performance between the two groups examined are presented in Table 3. The study findings indicated that a greater proportion of adolescents with IA had either unexcused school absences ($p = 0.004$) or had ceased attending school ($p = 0.002$) as compared to their non-IA peers. Moreover, among those remaining enrolled in school, a greater proportion of adolescents with IA had poor academic performance (45.3 vs. 20.9%, $p = 0.001$). In addition, the proportion of adolescents with IA engaging in extracurricular activities was significantly lower (54.7 vs. 88.4%, $p = 0.0001$) than their non-IA counterparts.

The proportion of adolescents engaging in high-risk behaviors was greater among adolescents with IA (32.6 vs. 0%, $p < 0.0001$). Furthermore, emotional problems were more frequently detected among adolescents with IA (84.9 vs. 20.9%, $p < 0.0001$). Moreover, the proportion of adolescents with comorbid psychiatric conditions, including depression ($p = 0.022$), was greater among those with IA (52.3 vs. 14.0%, $p < 0.0001$). Approximately only half (53.5%) of adolescents with IA had insight of their dysfunctional Internet behaviors. Finally, approximately one-third (31.0%) of the adolescents with IA presented with clinical signs and symptoms of addiction (Table 3).

**TABLE 2**
**Comparison of Family Characteristics among Adolescents According to the Presence of IA (n = 129)**

| | Case Group (n = 86) n (%) | Control Group (n = 43) n (%) | Chi-Square *p* Value |
|---|---|---|---|
| Parents' marital status | | | |
| Married | 62 (72.1) | 39 (90.7) | 0.016 |
| Divorced | 22 (25.6) | 3 (7.0) | 0.012 |
| Deceased | 2 (2.3) | 1 (2.3) | 0.450* |
| Father's highest education | | | |
| Primary school | 14 (16.3) | 5 (11.6) | 0.482 |
| Secondary school | 42 (48.8) | 18 (41.9) | 0.454 |
| College/university | 30 (34.9) | 20 (46.5) | 0.201 |
| Mother's highest education | | | |
| Primary school | 24 (27.9) | 13 (30.2) | 0.783 |
| Secondary school | 30 (34.9) | 11 (25.6) | 0.285 |
| College/university | 32 (37.2) | 19 (44.2) | 0.445 |
| Presence of siblings | 59 (68.6) | 35 (81.4) | 0.124 |
| Within family relationships | | | |
| Harmonic | 12 (14.0) | 32 (74.4) | <0.0001 |
| Dysfunctional | 41 (47.7) | 0 (0.0) | <0.0001* |
| Problematic boundaries | 18 (20.9) | 0 (0.0) | 0.001* |
| Conflict | 15 (17.4) | 11 (25.6) | 0.277 |

* Fisher's exact test *p* value.

The multivariate logistic regression analysis conducted (Table 4) indicated that adolescent emotional problems, comorbid psychiatric conditions, and lack of extracurricular activities were independent predictors of IA. Specifically, IA among adolescents was significantly associated with emotional problems (AOR: 17.15; 95% CI: 6.04–48.23) and comorbid psychiatric conditions (AOR: 3.89; 95% CI: 1.19–12.70). In contrast, IA among adolescents was inversely associated with engagement in extracurricular activities (AOR: 0.15; 95% CI: 0.04–0.54).

## DISCUSSION

The present study assessed the association between personal, family, and academic characteristics, as well as the presence of comorbid mental health conditions, and IA among adolescents. The main study findings indicated that IA among adolescents is associated with dysfunctional and/or problematic family relationships. In addition, adolescents with IA are more likely to have poor academic performance, limited extracurricular activities, and engagement in high-risk behaviors. Finally, IA among adolescents is associated with the presentation of emotional problems and concomitant psychiatric conditions, including depression. In the present study, the striking majority (95.4%) of adolescents with IA were male. The elevated occurrence of IA among male adolescents has been also reported in other cultural contexts[13,26,27], and may be potentially attributed to both the differential frequency and nature of Internet use observed among this group[2,7]. Specifically, male adolescents engage more frequently in high-risk Internet behaviors, including accessing the Internet for the purposes of retrieving pornographic

**TABLE 3**
**Comparisons of Academic Performance, Behaviors, and Clinical Characteristics of Adolescents According to the Presence of IA (n = 129)**

| | Case Group (n = 86) n (%) | Control Group (n = 43) n (%) | Chi-square P-value |
|---|---|---|---|
| Academic performance | | | |
| Unexcused school absences | 15 (17.4) | 0 (0.0) | 0.004 |
| Dropped out | 14 (16.3) | 0 (0.0) | 0.002* |
| Failure/poor performance | 39 (45.3) | 9 (20.9) | 0.001 |
| Good performance | 23 (26.7) | 13 (30.2) | 0.848 |
| Excellent performance | 10 (11.6) | 21 (48.8) | <0.0001 |
| Activities and behaviors | | | |
| Extracurricular activities | 47 (54.7) | 38 (88.4) | 0.0001 |
| High-risk behaviors | 28 (32.6) | 0 (0.0) | <0.0001 |
| Clinical characteristics | | | |
| Emotional problems | 73 (84.9) | 9 (20.9) | <0.0001 |
| Comorbid conditions | 45 (52.3) | 6 (14.0) | <0.0001 |
| ADHD | 18 (20.9) | 6 (14.0) | 0.337 |
| Depression | 9 (10.5) | 0 (0.0) | 0.022* |
| Awareness of addiction | 46 (53.5) | 0 (0.0) | <0.0001 |
| Clinical symptoms of addiction | 27 (31.0) | 0 (0.0) | <0.0001 |

* Fisher's exact test *p* value.

**TABLE 4**
**Multivariate Logistic Regression Analyses for Identifying the Determinants of IA among Adolescents**

| | Intercept | Standard Error | Odds Ratio (95% CI) |
|---|---|---|---|
| Emotional problems | 2.84 | 0.53 | **17.15 (6.04–48.23)** |
| Comorbid conditions | 1.36 | 0.60 | **3.89 (1.19–12.70)** |
| Extracurricular activities | -1.89 | 0.65 | **0.15 (0.04–0.54)** |

materials and gambling practices[28]. As a result, adolescent males may be at an enhanced risk for developing IA secondary to the scope of the Internet sites accessed.

With respect to family status, the study findings indicated that adolescents with IA were significantly more likely to report dysfunctional and/or problematic familial relationships. Previous study findings have indicated that both family dynamics and parenting styles are proximally associated with the development of IA among minors[29,30]. In particular, parenting styles that are either unsupportive or confrontational[31,32] have been associated with the development of IA among adolescents. Moreover, adolescents' perception of dysfunctional intrafamilial relationships, as well as dissatisfaction with such relationships, has also been correlated with IA[33,34]. Hence, both the actual and perceived natures of familial relationships may affect IA development[35], particularly among adolescents residing in single-parent households[36].

Furthermore, problematic peer relations and consequent social isolation[17] have been associated with the development of IA among adolescents[37]. Through both the anonymity and lack of immediate contact secured by the Internet, adolescent Internet users may interact within frameworks perceived as emotionally safe[38] and create idealized identities, particularly with respect to role-playing games[39]. These novel, albeit fictitious, identities may potentially ease participation in online social networks, thus contributing to the consequent evasion from real-world relationships[40]. Therefore, utilization of the Internet for the purposes of game playing[41] and socialization may contribute to the development and further manifestation of IA[25].

In excess of half of the adolescents with IA were reported as having poor academic performance. Furthermore, more than one-sixth of the adolescents with IA had either unexcused school absences or had dropped out of school. Such marked determent in academic functioning may occur secondary to the time allotted to Internet use[6,42] and/or limited perceived connectivity to the school environment[32]. Therefore, IA may notably adversely affect the academic performance of adolescents.

Excessive Internet use, as well as IA, has also been associated with generalized dysfunctional social behavior[39], as attributed to low self-esteem, shyness, and social withdrawal[43]. Furthermore, it has been posited that IA may present with comorbidity between IA and psychiatric disorders, such as depression[23,24,25], obsessive-compulsive disorder[25], anxiety, and psychosis[43]. The concomitant presence of ADHD and IA has been reported among youth[42]. The present study findings indicated that ADHD occurred among IA adolescents more frequently as compared to their normal Internet-user counterparts. However, the enhanced likelihood of concomitant ADHD among IA adolescents reported in previous studies[44,45] was not confirmed in the present study. Moreover, previous evidence indicating that ADHD constitutes a significant predictor of IA among adolescents[20] was not confirmed in the investigation presented. However, this discrepancy may be due to the limited study population size examined.

In contrast, the present study indicated that adolescents with IA were significantly more likely to present with emotional problems, including depressive symptoms, as compared to their normal Internet-user counterparts. Furthermore, approximately 10.5% of adolescents with IA were observed to concomitantly present with depressive symptoms. This is in agreement with previous reports indicating that young adults who excessively use the Internet have an enhanced likelihood for presenting with suicidal ideation[22] and depression[23,32,36], particularly among males[46]. IA may have both internal and external adverse effects upon individual functioning[47]. Compromised social relationships and networks, as well as augmented social withdrawal, are often manifestations consequent to IA[43]. However, depression may also constitute an etiological factor of IA among adolescents[20]. While the present study findings indicate that a significant association exists between IA and emotional symptoms, the etiological direction of this association cannot be concretely established.

**Ref. 49 not cited in text. Where should it be placed?**

The strengths of the present study include that it is one of the few of its kind to assess the characteristics associated with IA among youth. All study participants were assessed by two independent examiners who were blinded to the study objectives and, thus, it is upheld that a misclassification bias was deterred. One of the study limitations was that the control group utilized consisted of a clinical sample. As a result, the ability to extrapolate the study findings to the general population may be compromised. In addition, due to the potential presence of confounding factors, the present study does not allow for the determination of the causal association between psychiatric conditions and IA development.

In conclusion, the present study findings indicated that adolescents with IA had an augmented likelihood to report dysfunctional familial relationships, poor academic performance, and engage in high-risk behaviors. In addition, IA among adolescents was significantly associated with comorbid psychiatric conditions, including depression. Therefore, IA among adolescents is associated with deterred familial functions, poor academic performance, engagement in high-risk behaviors, and enhanced risk for depression. Prospective cohort studies are necessary in order to determine the risk factors for the development of IA among youth.
